# Editorial: *In-vivo* targeting of nuclear DNA with radioactive copper-64 ions

**DOI:** 10.3389/fmed.2023.1334294

**Published:** 2023-11-23

**Authors:** Bianca Gutfilen, Adriano Duatti

**Affiliations:** ^1^Laboratory of Labeling Cells and Molecules, Federal University of Rio de Janeiro, Rio de Janeiro, Brazil; ^2^Department of Chemical and Pharmaceutical Sciences, University of Ferrara, Ferrara, Italy

**Keywords:** copper-64, nuclear DNA, positron emission tomography, radionuclide therapy, theranostics, Auger electrons, linear energy transfer (LET), DNA targeting

## Copper-64 in cancer

Copper-64 decays through the emission of positrons and a cascade of low-energy Meitner-Auger electrons. Therefore, it can be used for both positron emission tomography (PET) imaging and cancer therapy by taking advantage of the high linear-energy-transfer of Meitner-Auger electrons.

After the introduction of efficient methods for producing ^64^Cu by low-energy medical cyclotrons, interest in the use of ^64^Cu radiopharmaceuticals has rapidly grown and the first ^64^Cu imaging agent [[^64^Cu]Cu-DOTATATE] has received marketing authorization. Copper-64 is produced in a buffered aqueous solution under the simple chemical form of its chloride salt [^64^Cu][CuCl_2_] (1– 3).

Perhaps because it was unpredicted, the observation that [^64^Cu][Cu]^2+^ ions are selectively taken up by many types of cancer (4–11) has been largely ignored by the scientific community. However, subsequent research and Phase I/II clinical studies in patients have unequivocally demonstrated that simple [^64^Cu][Cu]^2+^ ions can be successfully used in positron emission tomography for the early diagnosis of glioblastoma and prostate cancer (9–11). On this ground and considering the persistent lack of an effective therapy for glioblastoma multiforme, interest in the theranostic use of ^64^Cu has been focused on the treatment of this deadly form of brain tumor. Therefore, further clinical trials have been designed to evaluate the efficacy of ionic [^64^Cu][Cu]^2+^ against glioblastoma. Based on the encouraging results obtained in these clinical trials, the European Medicine Agency (EMA) has granted “orphan drug” status to [^64^Cu][CuCl_2_] administered intravenously as an aqueous buffered solution for the treatment of glioblastoma (12).

It is reasonable to expect that ^64^Cu can achieve its potent therapeutic effect only when short-range Meitner-Auger electrons are emitted in the neighborhoods of the nucleus. This implies that ^64^Cu ions should be able to cross the nuclear membrane and come into close contact with the genetic material of tumor cells. This hypothesis is also supported by the observation that copper plays a critical role in the signal transduction pathway that regulates tumor cell proliferation and tumor growth. Many biological experiments conducted on isolated cells have provided convincing experimental evidence that [^64^Cu][Cu]^2+^ ions can translocate into the cell nucleus and these findings are in agreement with the results of many preclinical studies performed on animal models (13). However, perhaps the most irrefutable evidence that copper (II) ions can penetrate the nuclear region was obtained by purely physical methods. The ability to accurately determine the subcellular localization of a radionuclide depends on the spatial resolution of the experimental method used. Usually, measuring the radiation emission does not allow to reach the spatial precision necessary to determine the exact subcellular localization of a radioactive species, which always falls within the micrometric range. Recently it has been possible to detect the absorption of copper by the cell nucleus in cancerous cells using sophisticated spectrometric techniques with very high spatial resolution. Specifically, Time-of-Flight Secondary Ion Mass Spectrometry (ToF-SIMS) combined with delayed extraction has been successfully applied to probe copper localization in fixed MDA-MB-231 breast cancer cells providing subcellular resolution of approximately 350–400 nm. Notably, experimental findings revealed that copper is rather homogeneously distributed between the nucleoplasm and the nuclear membranes (14–17).

## Scope of the Research Topic and main results

The aim of the Research Topic ‘*In-vivo targeting of nuclear DNA with radioactive copper-64 ions*' (https://www.frontiersin.org/research-topics/34471/in-vivo-targeting-of-nuclear-dna-with-radioactive-copper-64-ions) was to explore further the interaction of cupric ions and copper compounds with nuclear DNA of cancerous cells and to evaluate the therapeutic efficacy of these species when labeled with the radionuclide ^64^Cu. Although a limited number of articles have been submitted and published, their scientific relevance is remarkable.

In their paper, Khosravifarsani et al. applied a classical bifunctional approach by tethering the radioactive complex [^64^Cu]Cu-NOTA, where NOTA is the macrocyclic chelator 2,2′,2″,2^‴^-(1,4,7,10-tetraazacyclododecane-1,4,7,10-tetrayl) tetraacetic acid, to a novel terpyridine-platinum (TP) compound as G-4 quadruplex DNA binder. The resulting conjugate compound [^64^Cu]Cu-NOTA-C3-TP can behave as both a chemotherapeutic and a radiotheranostic drug. The biological properties of this complex have been investigated both on isolated cancer cells and in preclinical mouse models.

Serban et al. conducted an extensive study on various types of cultured cancer cells focused on measuring the damage produced by the emission of Meitner-Auger electrons when these cells were treated with [^64^Cu]CuCl_2_. The effect of radiation emission on cells was quantified by measuring radioisotope uptake and retention, cell viability and death, DNA damage, oxidative stress, and the expression of 84 stress genes. The strongest cytotoxic effects of the radioisotope were observed in colon carcinoma HCT116 cells, where a substantial decrease in the number of metabolically active cells and an increased DNA damage and oxidative stress were registered. The stress gene expression experiments highlighted the activation of both death and repair mechanisms (apoptosis, necrosis/necroptosis or autophagy, and cell cycle arrest, nucleotide excision repair, antioxidant, and hypoxic responses) in these cells after radiation exposure.

A third paper by Carrasco-Hernandez et al. nicely complemented the work of Serban et al. on the quantification of the therapeutic potential and DNA damage of ^64^Cu incorporated into the DNA of mammalian cells using a theoretical model based on Monte Carlo track-structure simulations. Results suggest the ability of ^64^Cu to induce a lethal effect when incorporated into the DNA genome and the initial activity per cell calculated to cause lethal damage can be used to estimate the total activity necessary to administer in a group of cells or tissue for targeted radionuclide therapy (TRT).

## Conclusions

Undoubtedly, the ion [^64^Cu][Cu^2+^] constitutes a new example of a radiolabeled monatomic species that adds to the list of other radioactive ions like [^131^I][I^−^], [^201^Tl][Tl^+^], and [^82^Rb][Rb^+^], which have played a fundamental role in the development of nuclear medicine (18). Similarly to the iodide anion, ionic copper can be used both as a precursor for the preparation of radiopharmaceuticals, in which the ion is linked to a suitable pharmacophore, and as a free ion since it has revealed to possess intrinsic biological properties that make it an potential theranostic agent especially for cancer diagnostics and therapy. In this regard, it can be said that one of the most notable biological characteristics shown by the [^64^Cu][Cu^2+^] ion is that it is transported within the nuclear region of cancerous cells where it can it interact with their genetic material. From this point of view, the copper ion can be considered as one of the few *in vivo* DNA markers. The results of experiments on glioblastoma therapy with ionic Cu-64, in which nuclear localization is observed only in abnormal cells and not in healthy cells, support the hypothesis that nuclear translocation of [^64^Cu][Cu^2+^] may only occur in malignant cells where the distribution of genetic material within the nuclear region has been completely altered. In the future, it will be necessary to conduct other experimental tests to have a comprehensive representation of the behavior of the Cu-64 ion inside tumor cells and to understand whether these properties can be exploited for treating cancer. However, the results from clinical studies conducted with ionic copper, together with those where copper is used as a precursor for the labeling of biologically active molecules, clearly indicate that this radionuclide will play a crucial role in the development of theranostic nuclear medicine.

## Author contributions

BG: Conceptualization, Writing – original draft, Writing – review & editing. AD: Conceptualization, Writing – original draft, Writing – review & editing.
